# Impacts of climate change on *Capparis spinosa* L. based on ecological niche modeling

**DOI:** 10.7717/peerj.5792

**Published:** 2018-10-16

**Authors:** Uzma Ashraf, Muhammad N. Chaudhry, Sajid R. Ahmad, Irfan Ashraf, Muhammad Arslan, Hassaan Noor, Mobeen Jabbar

**Affiliations:** 1 Department of Environmental Sciences and Policy, Lahore School of Economics, Lahore, Lahore, Punjab, Pakistan; 2 College of Earth and Environmental Sciences, University of the Punjab, Lahore, Punjab, Pakistan; 3 Strategic Policy Unit, Lahore Development Authority, Lahore, Punjab, Pakistan; 4 Environmental Biotechnology Department, Helmholtz Center for Environmental Research, Leipzig, Germany

**Keywords:** *Capparis spinosa*, Climate change, Ecological niche modeling, Potential distribution

## Abstract

Recent changes in climate are transforming the situation of life on Earth, including impacting the conservation status of many plant and animal species. This study aims to evaluate potential impacts of climate change on a medicinal plant that is known to be heat-tolerant, *Capparis spinosa* L. We used ecological niche modeling to estimate current and future potential distributions for the species, considering two emissions scenarios and five climate models for two time periods (2050 and 2070). The results in terms of areal coverage at different suitability levels in the future were closely similar to its present-day distribution; indeed, only minor differences existed in highly suitable area, with increases of only 0.2–0.3% in suitable area for 2050 and 2070 under representative concentration pathway 4.5. Given that climate-mediated range shifts in the species are expected to be minor, conservation attention to this species can focus on minimizing local effects of anthropogenic activity.

## Introduction

In recent years, climate change has been established as a clear and emerging global process, and adverse impacts on biological elements have been reported worldwide (Bellard et al., 2012). Climate change effects have been documented for both human populations and biodiversity (Chang et al., 2015); examples of the latter include pest and disease outbreaks (Woods, Coates & Hamann, 2005), temporal reproductive isolation (Lowry & Willis, 2010), and changes in species’ distribution and phenology (Peterson et al., 2004). The situation is expected to become more dramatic as the pace and magnitude of environmental changes quicken (Meehl et al., 2007), and may prove to be more adverse in developing countries as compared to developed countries (IPCC, 2014).

*Capparis spinosa*, known commonly as flinders rose or caper bush, is an evergreen shrub that prefers dry heat and intense sunlight. The drought- and salt-tolerant nature of the species allows it to persist in a wide range of habitats, even on nutrient-poor, sandy, and gravelly soils (Özkahraman, 1997; Sakcali, Bahadir & Ozturk, 2008). The species is apparently native to dry regions of western and central Asia; however, long ago it spread to the Mediterranean Basin, southern Europe, North and East Africa, Madagascar, Australia, and Oceania (Inocencio et al., 2002; Chedraoui et al., 2017). Although sometimes considered a weed, it has a long history as an archaeophyte (Jiang et al., 2007). Immature flower buds, unripe fruits, and shoots are consumed as foods or condiments. Flower buds, fruits, seeds, shoots, and bark of roots were traditionally used for pharmacological purposes, especially for treating rheumatism (Jiang et al., 2007; Renfrew, 1973). This plant is widely appreciated for its medicinal properties, including curative properties for a variety of health problems, particularly diseases of the spleen, kidney, and liver (Sakcali, Bahadir & Ozturk, 2008; Khatib et al., 2016). These properties apparently derive from compounds such as rutin, carotenoid, vitamin C, and tocopherols (Khatib et al., 2016). This plant is also potentially important in combating desertification and minimizing soil erosion, thanks to its drought-resistant natural history (Sakcali, Bahadir & Ozturk, 2008).

Ecological niche modeling (ENM) and the related ideas of species distribution modeling and habitat suitability modeling have gained popularity since they allow evaluation of potential geographic distributions of species under scenarios of climate change (Huntley et al., 2008). The approach employs information on environmental variables associated with ecological niche constraints on a species in relation to future distributions of those conditions as approximated by climate model outputs (Araújo & Peterson, 2012). The result of these models is the coarse-resolution correlative model of the ecological niche, which makes geographic predictions of the distributional potential of species under present and future conditions. These models are evaluated for predictive ability based on careful testing with independent occurrence datasets.

Although several studies have examined structural and functional traits, phenotypic plasticity, and genetic variation in this species (Saifi, Ibijbijen & Echchgadda, 2011; Chedraoui et al., 2017), little information is available on its actual and potential geographic distribution and climate change sensitivity. The present study therefore aims to (1) characterize the species’ ecological niche with respect to climate, and (2) estimate present and future potential distributional areas of *C. spinosa* worldwide.

## Materials and Methods

### Study area and datasets

**Figure 1:**
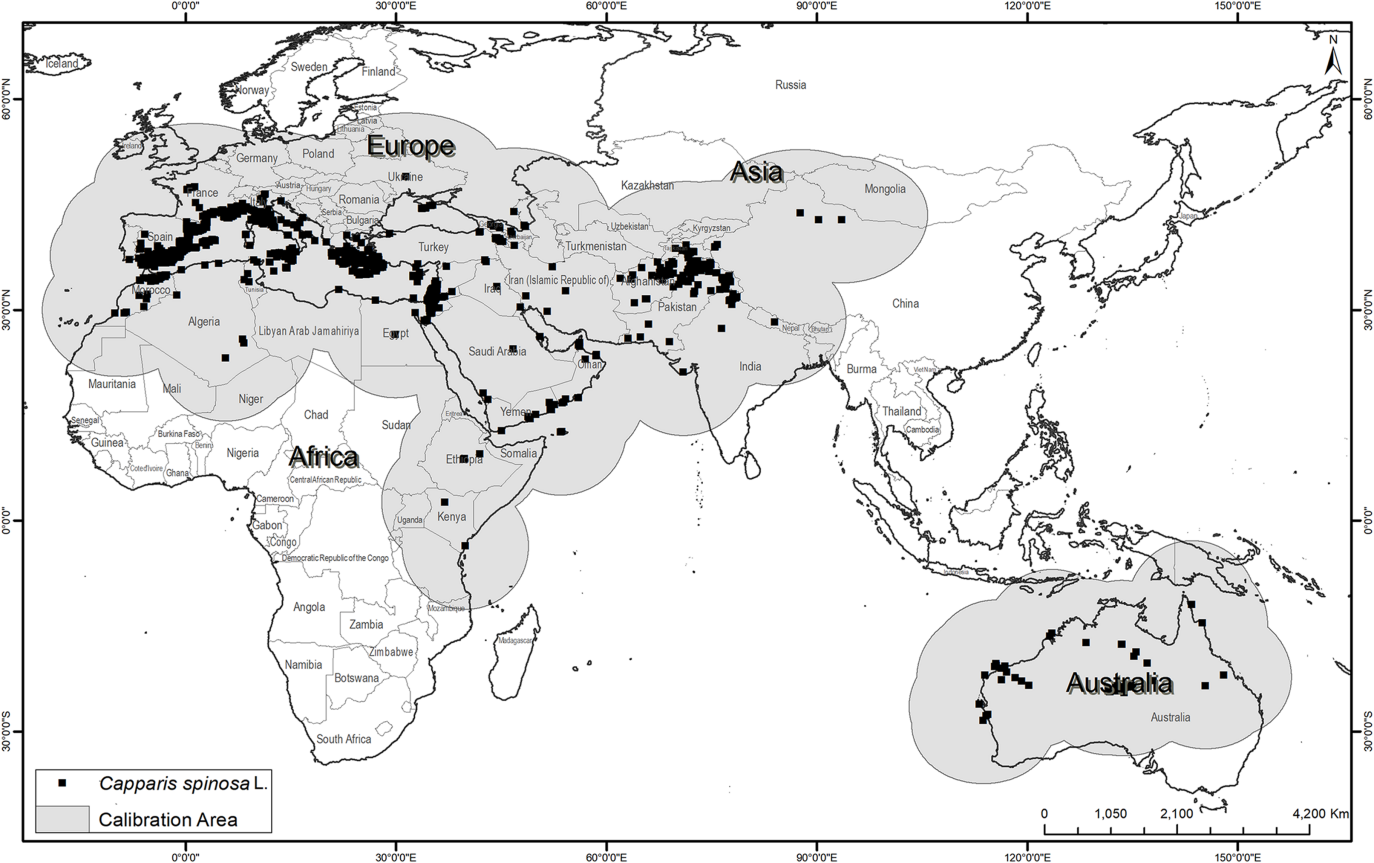
Known occurrences of *Capparis spinosa* L. worldwide used in model calibration.

Occurrence data were obtained from the Global Biodiversity Information Facility website (https://www.gbif.org), with an initial total of 6,371 occurrence points corresponding to 15 infraspecies (Fig. 1; https://doi.org/10.15468/dl.cu3tvo). These data were checked rigorously for georeferencing errors and spatial biases. Outliers and botanical garden-based points were removed through careful assessment of data source and consultation of record metadata, leaving 5,508 points for further analysis. To estimate the area (**M**) that has been accessible to the species over relevant time periods (Barve et al., 2011), which should be used as the model calibration area, we applied a buffer of 1,000 km around all of the known occurrence points (Fig. 1) (Barve et al., 2011). In light of the relatively subtle differences between present and future potential distributions, and considering the problems associated with model transfers to other regions (Yates et al., 2018), we have opted not to transfer our models globally, but rather to explore implications of climate change within the **M** region only.

Environmental variables were obtained from the WorldClim climate data archive (http://www.worldclim.org) (Fick & Hijmans, 2017), in the form of 19 bioclimatic data layers summarizing potentially relevant climate dimensions at 2.5′ spatial resolution. Parallel bioclimatic data layers were obtained for five downscaled general circulation model (GCM) outputs at CCAFS Climate (http://www.ccafs-climate.org). We considered 2050 and 2070 as future time slices, under two emission scenarios: representative concentration pathways (RCPs) 4.5 and 8.5. As such, we explored 5 GCMs × 2 time periods × 2 RCPs = 20 future transfers of models for the species.

### Processing

Occurrence data were rarefied to leave no pair of points closer than five km using SDMTools (Brown, 2014) in ArcGIS, reducing biases related to spatial autocorrelation. This spatial filtering reduced the number of occurrence points still further to 1,287. We divided these data into two equal parts at random to permit model calibration and evaluation. We also calculated Pearson correlation coefficients among environmental variables (Table S1) to avoid using non-independent variables in model calibration. One from each pair of variables showing high correlations (i.e., *r* > 0.9) is eliminated (Paulo et al., 2015; Bemmels et al., 2016). Variable choice from each pair for retention is based on its variable importance in preliminary model runs (Table S1). Variables removed were annual mean temperature, minimum temperature of coldest month, temperature annual range, mean temperature of warmest quarter, and precipitation of wettest and driest quarters. We further removed mean temperature of wettest and driest quarters, and precipitation of warmest and coldest quarters, as they present odd spatial artifacts (Ribeiro et al., 2017). Variables used as predictors in final models are presented in Table 1.

**Table 1:** Percentage and permutation importance of variables for prediction of *Capparis spinosa* L.

Variables	Percent importance (%)	Permutation importance (%)
Mean temperature of coldest quarter	26.96	10.20
Annual precipitation	10.37	13.07
Precipitation of wettest month	1.15	2.57
Precipitation of driest month	6.05	2.20
Precipitation seasonality	0.83	0.77
Mean diurnal range	29.86	22.95
Isothermality	10.04	4.15
Temperature seasonality	9.50	29.90
Maximum temperature of warmest month	5.24	14.19

MaxEnt (version 3.3.4k) (Phillips & Dudík, 2008) was used for calibrating models, as well as for assessing the importance of environmental variables (see above). Maxent settings were 5,000 iterations, 10 random replicate analyses, and 10% test sample size; all other options were left on default. For evaluation of model predictions, we used partial receiver operating characteristic (ROC) approaches (Peterson, Papeş & Soberón, 2008). Partial ROC statistics were calculated using online the Niche Toolbox site, http://shiny.conabio.gob.mx:3838/nichetoolb2/, with 1,000 replicates and *E* = 0.1% (Peterson, Papeş & Soberón, 2008). Final models were calibrated based on present-day conditions and transferred to each of the 20 future-climate datasets.

Finally, we developed binary models based on relationships between the calibration points and the raw model outputs. Threshold values were decided on the basis of the calibration dataset, identifying the highest thresholds corresponding to 0%, 5%, and 10% omission error rates. We calculated change in high, moderate, and low suitability areas in all future transfers as follows: 0–0.09 threshold value (*E* = 0–5%) as low suitability area, 0.09–0.19 threshold value (*E* = 5–10%) as moderate suitability area, and above 0.19 threshold value (*E* ≥ 10%) as highly suitable area. Future-transfer maps were thresholded based on the same criteria as the present-day models, and percentages of area were calculated to facilitate comparisons.

## Results

The final model performed better than random expectations, based on the random independent subset of occurrence data (area under the curve (AUC) ratio 1.85, *P* < 0.001) (Fig. S1). Table 1 summarizes relative contributions of environmental predictor variables to the model. The most important predictor variable was mean diurnal range (Table 1), which is the difference between mean maximum monthly temperature and mean minimum monthly temperature. Mean temperature of the coldest quarter was the second most important factor for this species; the least important environmental variable was precipitation seasonality. Indeed, precipitation-related variables in general showed minimal contributions to model quality.

**Figure 2:**
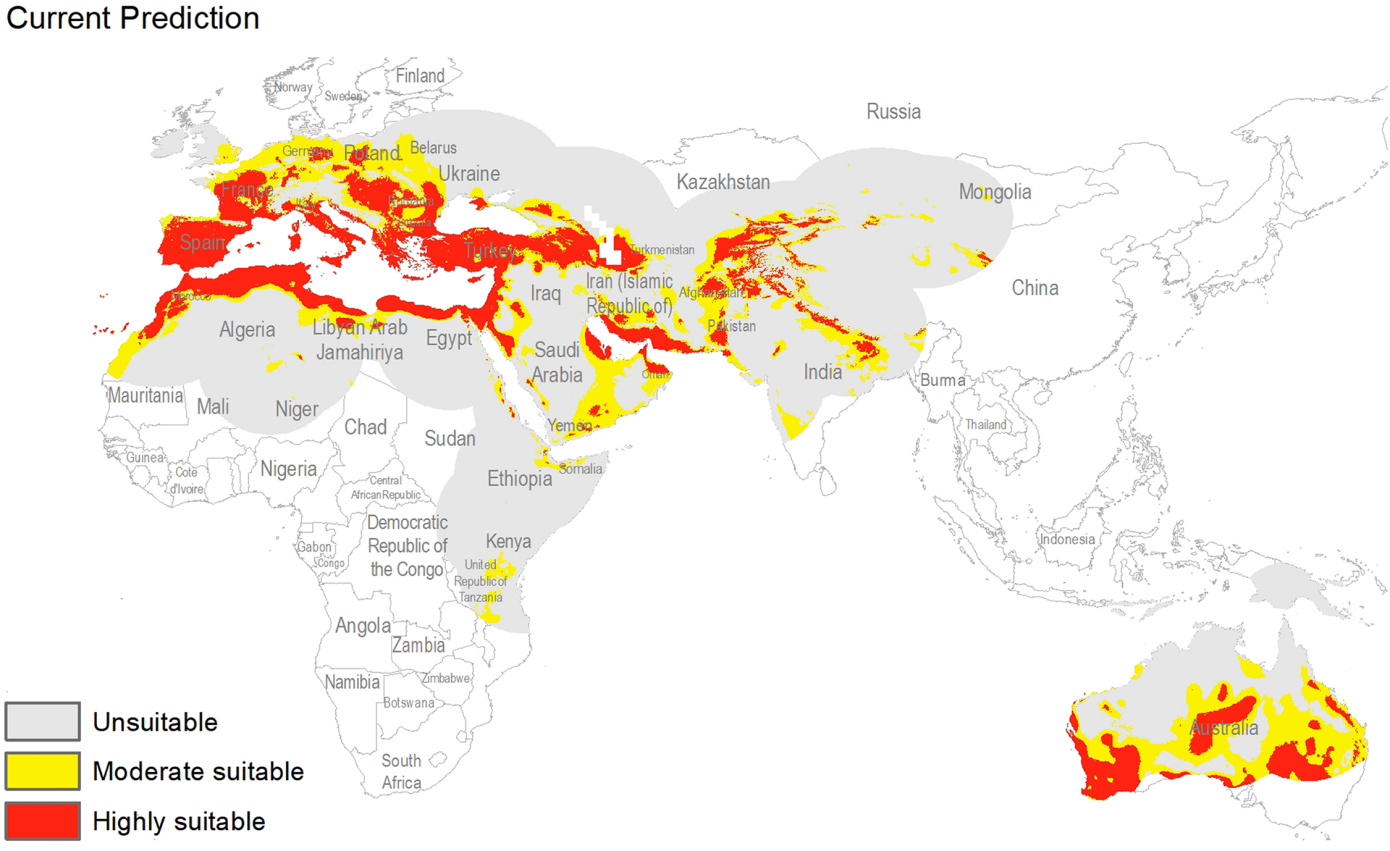
Potential distribution of *Capparis spinosa* L. under present day climatic conditions.

**Figure 3:**
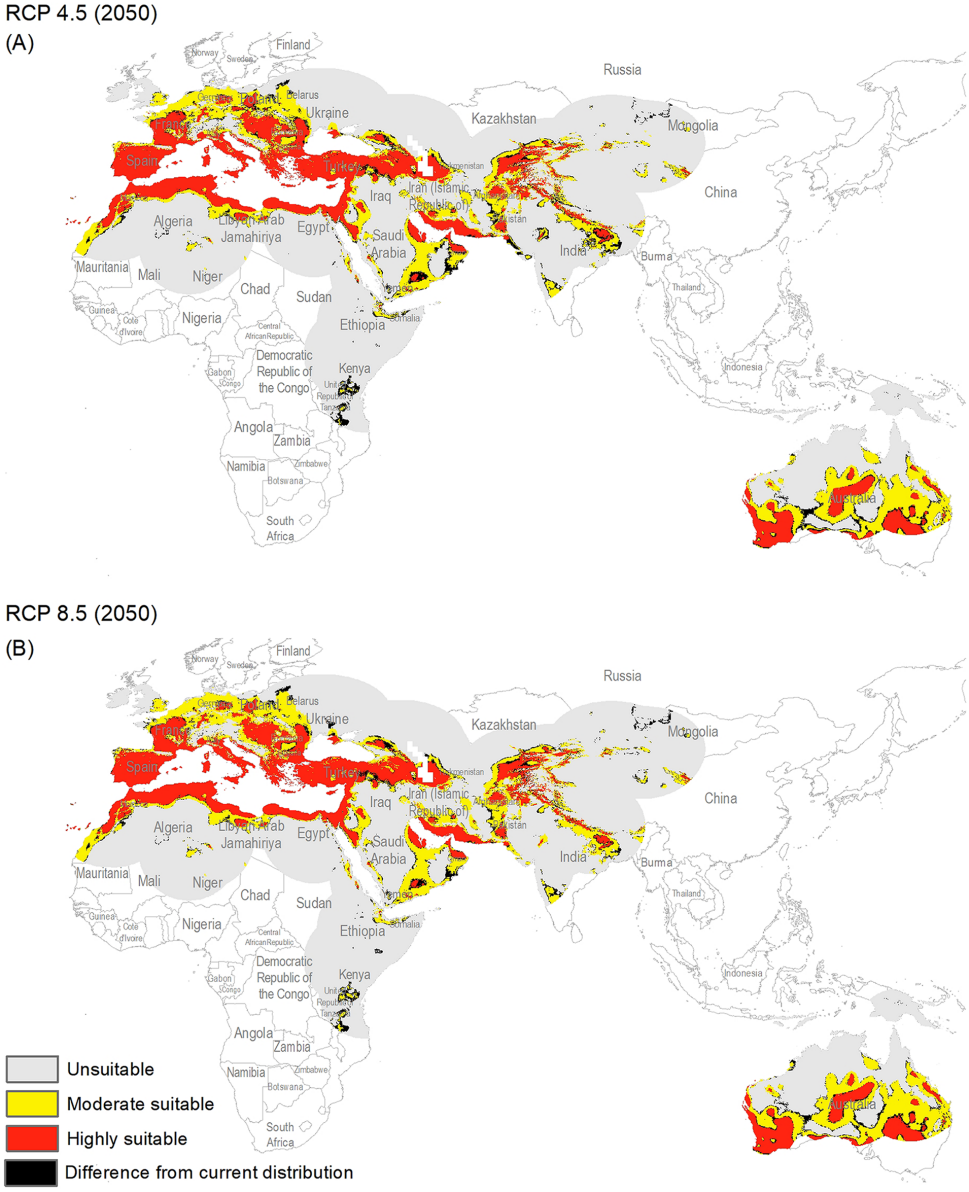
Maps of the potential distribution of *Capparis spinosa* L. under 2050 climate conditions ((A) map shows the average prediction for RCP 4.5, (B) map shows the average prediction for RCP 8.5).

**Figure 4:**
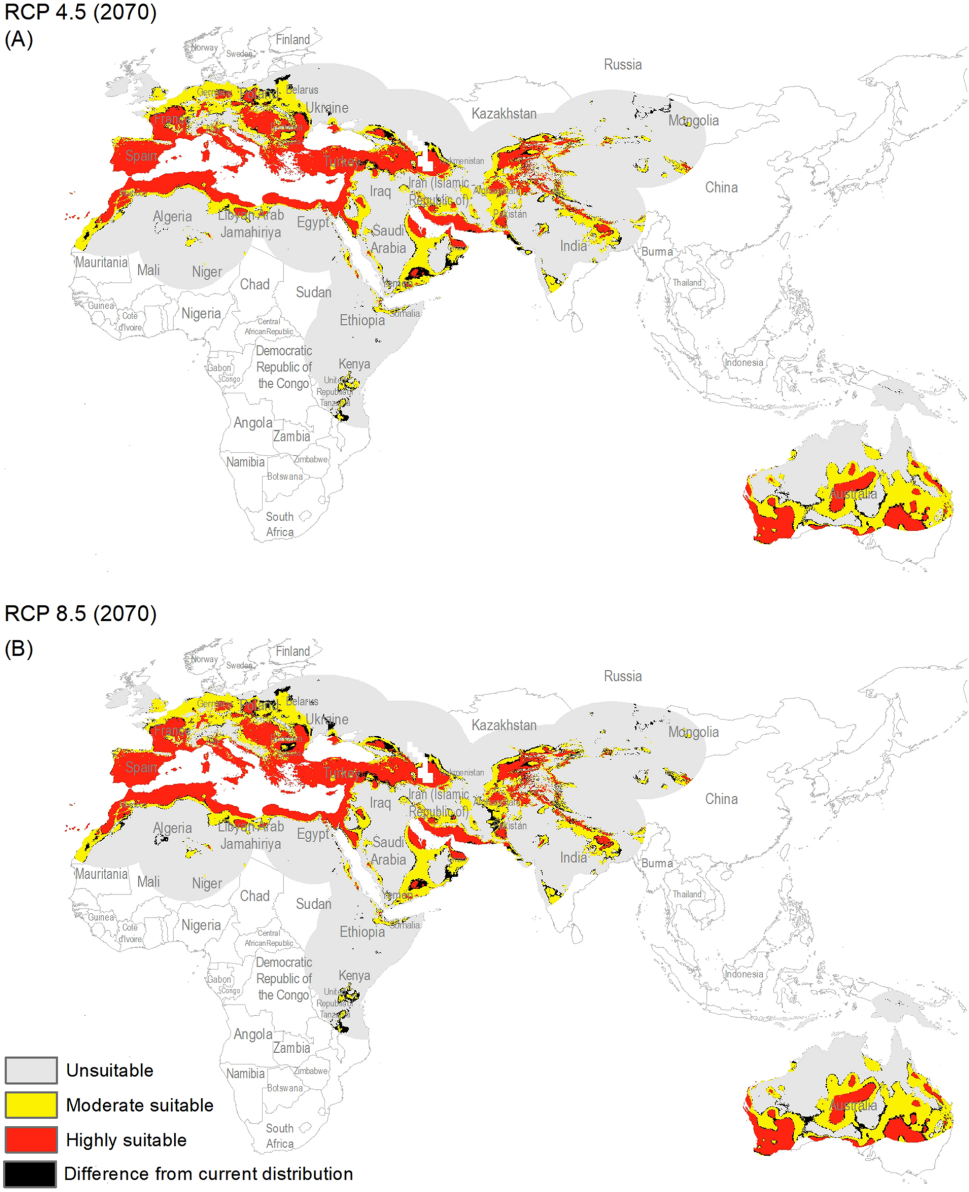
Maps of the potential distribution of *Capparis spinosa* L. under 2070 climatic conditions ((A) map shows the average prediction for RCP 4.5, (B) map shows the average prediction for RCP 8.5).

The potential distribution for the present day identified by our models indicates that this species is able to maintain populations in arid, semi-arid, and Mediterranean climate regions, including parts of Asia, Africa, Europe, and Australia (Fig. 2), with highly suitable conditions covering 16% of the study area. For future model transfers, results in term of areal coverage (Table 2) at different suitability levels were closely similar to present-day patterns (Figs. 3 and 4). Overall, only minor differences existed in highly suitable area, with increases of 0.2–0.3% in suitable area under RCP 4.5 (2070) and RCP 4.5 (2050), respectively (Table 2; Figs. 3 and 4). Indeed, under all scenarios, areal coverage by suitable conditions changed only negligibly between present-day and future conditions.

**Table 2:** Area calculations for two representative pathway (RCPs) for two time slices for the potential distributional area of *Capparis spinosa* L.

Classification	Current (%)	2050 (%)		2070 (%)	
		RCP 4.5	RCP 8.5	RCP 4.5	RCP 8.5
Unsuitable	66.94	66.71	67.36	66.69	66.87
Moderately suitable	17.24	17.21	16.84	17.14	16.98
Highly suitable	15.83	16.08	15.81	16.17	16.15

## Discussion

Model performance evaluation is a key step in determining the accuracy of ecological niche models and the resulting distributional predictions (Peterson, Papeş & Soberón, 2008). Maxent models often have high accuracy in comparison to other modeling algorithms (Elith et al., 2006; Phillips, Anderson & Schapire, 2006; Chalghaf et al., 2016), and indeed our model performed well, anticipating independent data subsets much better than null expectations. Although transfers of niche models to future conditions require additional assumptions (Peterson, Cobos & Jiménez-García, 2018), our tests at least confirm the ability of our model predictions to anticipate present-day distributional phenomena.

For both current and future time periods, countries having Mediterranean climates, with hot and dry summers (drought conditions) were predicted as highly suitable for this species, such as Portugal, Spain, France, Morocco, Italy, Greece, Hungary, Turkey, Bulgaria, Armenia, and parts of Tunisia, Egypt, Libya, Algeria, Afghanistan, Tajikistan, Pakistan, Yemen, and Australia. Countries at higher latitudes were predicted to hold unsuitable habitat for this species: for example, Latvia, Ukraine, Belarus, Lithuania, Kazakhstan, Turkmenistan, China, Ireland, and UK. Variable contributions also revealed that hot and dry climates appear to be key for this species, as precipitation was much less important than temperature in model calibration.

The potential distribution of *C. spinosa* showed stability in some parts of the world, and mild expansion in others, in the face of changing climates into the future. Being a xerophytic shrub, the species offers several ecosystem services regarding erosion control, and shows remarkable adaptability toward harsh environments (Chedraoui et al., 2017). The species grows mainly in arid and semi-arid environments in tropical regions, and thus can be used to combat desertification and soil erosion (Sakcali, Bahadir & Ozturk, 2008). It is also considered an important plant both economically and medicinally, and has been used in Chinese, Ayurvedic, Siddha, and Unani medicine from many centuries (Faran, 2014). For instance, its root bark has analgesic, anthihaemorrhoidal, depurative, anthelmintic, and tonic properties, and has been used to treat gastrointestinal infections (Faran, 2014).

This plant is native to Asia, and has a long history farther west, in Mediterranean Europe; it is invasive in North America (Chedraoui et al., 2017). The medicinal properties of this species are appreciated across its native range. Our models indicate that climate change poses few or no direct challenges to its persistence. Anthropogenic pressures such as human population pressure and associated land-use change and livestock grazing, may reduce its populations, leading to soil erosion and desertification, which would require restoration and rehabilitation of land areas toward conservation. Chedraoui et al. (2017) presented a comprehensive review of the social, economical, and ecological properties of *C. spinose*; the review also encouraged studies on this species in relation to climate change in the Mediterranean region, which is presented in this contribution.

## Conclusion

In this study, ENM was used to assess the likely current and future potential distribution of *C. spinosa*, under different emissions scenarios for two time periods (2050 and 2070). These explorations revealed that, under future climatic scenarios, impacts of these changes on the distributional potential of *C. spinosa* will be minimal. Temperature places strongest constraints on populations of the species. As this species can grow in hot and arid environments, this species may increase in the arid, semi-arid, and Mediterranean climate regions in the future.

## Supplemental Information

10.7717/peerj.5792/supp-1 Supplemental Information 1 A folder containing M shapefile and the final species sighting datasets that used for calibration and evaluation.

10.7717/peerj.5792/supp-2 Supplemental Information 2 Partial AUC results. The distribution is of a 50% bootstrap resampling (1000 replicate samples), and the critical value is 1.0, far to the left of the graph.

10.7717/peerj.5792/supp-3 Supplemental Information 3 Pearson correlation coefficient between environmental layers.

## Additional Information and Declarations

### Data Availability

The following information was supplied regarding data availability: The raw data are provided in the Supplemental Files.

## References

[ref-1] Araújo MB, Peterson AT (2012). Uses and misuses of bioclimatic envelope modeling. Ecology.

[ref-2] Barve N, Barve V, Jiménez-Valverde A, Lira-Noriega A, Maher SP, Peterson AT, Soberón J, Villalobos F (2011). The crucial role of the accessible area in ecological niche modeling and species distribution modeling. Ecological Modelling.

[ref-3] Bemmels JB, Title PO, Ortego J, Knowles LL (2016). Tests of species-specific models reveal the importance of drought in postglacial range shifts of a Mediterranean-climate tree: insights from integrative distributional, demographic and coalescent modelling and ABC model selection. Molecular Ecology.

[ref-4] Bellard C, Bertelsmeier C, Leadley P, Thuiller W, Courchamp F (2012). Impacts of climate change on the future of biodiversity. Ecology Letters.

[ref-5] Brown JL (2014). SDMtoolbox: a python-based GIS toolkit for landscape genetic, biogeographic and species distribution model analyses. Methods in Ecology and Evolution.

[ref-6] Chang XY, Chen BM, Liu G, Zhou T, Jia XR, Peng SL (2015). Effects of climate change on plant population growth rate and community composition change. PLOS ONE.

[ref-7] Chalghaf B, Chlif S, Mayala B, Ghawar W, Bettaieb J, Harrabi M, Benie GB, Michael E, Salah AB (2016). Ecological niche modeling for the prediction of the geographic distribution of cutaneous *leishmaniasis* in Tunisia. American Journal of Tropical Medicine and Hygiene.

[ref-8] Chedraoui S, Abi-Rizk A, EI-Beyrouthy M, Chalak L, Ouaini N, Rajjou L (2017). *Capparis spinosa* L. in a systematic review: a xerophilous species of multi values and promising potentialities. Frontiers in Plant Science.

[ref-31] Elith J, Graham CH, Anderson RP, Dudík M, Ferrier S, Guisan A, Hijmans RJ, Huettmann F, Leathwick JR, Lehmann A, Li J, Lohmann LG, Loiselle BA, Manion G, Moritz C, Nakamura M, Nakazawa Y, Overton JMM, Peterson AT, Phillips SJ, Richardson K, Scachetti-Pereira R, Schapire RE, Soberón J, Williams S, Wisz MS, Zimmermann NE (2006). Novel methods improve prediction of species’ distributions from occurrence data. Ecography.

[ref-9] Faran M (2014). Capparis spinosa—the plant on the wall: in medicinal and aromatic plants of the Middle-East.

[ref-10] Fick SE, Hijmans RJ (2017). WorldClim 2: new 1-km spatial resolution climate surfaces for global land areas. International Journal of Climatology.

[ref-11] Huntley B, Collingham YC, Willis SG, Green RE (2008). Potential impacts of climatic change on European breeding birds. PLOS ONE.

[ref-12] Inocencio C, Alcaraz F, Calderón F, Obón C, Rivera D (2002). The use of floral characters in *Capparis* sect. *Capparis* to determine the botanical and geographical origin of capers. European Food Research and Technology.

[ref-13] Intergovernmental Panel on Climate Change (IPCC) (2014). Climate Change 2014—impacts, adaptation and vulnerability: regional aspects.

[ref-14] Jiang HE, Li X, Ferguson DK, Wang YF, Liu CJ, Li CS (2007). The discovery of *Capparis spinosa L*. (Capparidaceae) in the Yanghai Tombs (2800 years b.p.), NW China, and its medicinal implications. Journal of Ethnopharmacology.

[ref-15] Khatib M, Pieraccini G, Innocenti M, Melani F, Mulinacci N (2016). An insight on the alkaloid content of *Capparis spinosa* L. root by HPLC-DAD-MS, MS/MS and ^1^H qNMR. Journal of Pharmaceutical and Biomedical Analysis.

[ref-16] Lowry DB, Willis JH (2010). A widespread chromosomal inversion polymorphism contributes to a major life-history transition, local adaptation, and reproductive isolation. PLOS Biology.

[ref-17] Meehl GA, Covey C, Taylor KE, Delworth T, Stouffer RJ, Latif M, McAvaney B, Mitchell JFB (2007). The WCRP CMIP3 multimodel dataset: a new era in climate change research. Bulletin of the American Meteorological Society.

[ref-18] Özkahraman I (1997). Caper.

[ref-19] Peterson AT, Cobos ME, Jiménez-García D (2018). Major challenges for correlational ecological niche model projections to future climate conditions. Annals of the New York Academy of Sciences.

[ref-20] Peterson AT, Martínez-Meyer E, González-Salazar C, Hall PW (2004). Modeled climate change effects on distributions of Canadian butterfly species. Canadian Journal of Zoology.

[ref-21] Peterson AT, Papeş M, Soberón J (2008). Rethinking receiver operating characteristic analysis applications in ecological niche modeling. Ecological Modelling.

[ref-22] Phillips SJ, Dudík M (2008). Modeling of species distributions with Maxent: new extensions and a comprehensive evaluation. Ecography.

[ref-23] Phillips SJ, Anderson RP, Schapire RE (2006). Maximum entropy modeling of species geographic distributions. Ecological Modelling.

[ref-24] Paulo JA, Palma JHN, Gomes AA, Faias SP, Tomé J, Tomé M (2015). Predicting site index from climate and soil variables for cork oak (*Quercus suber L*.) stands in Portugal. New Forests.

[ref-25] Renfrew JM (1973). Palaeoethnobotany: The Prehistoric Food Plants of the Near East and Europe.

[ref-26] Ribeiro V, Peterson AT, Werneck FP, Machado RB (2017). Ecological and historical views of the diversification of *Geositta* miners (Aves: Furnariidae: Sclerurinae). Journal of Ornithology.

[ref-27] Saifi N, Ibijbijen J, Echchgadda D (2011). Genetic diversity of caper plant (*Capparis ssp*.) from North Morocco. Journal of Food, Agriculture & Environment.

[ref-28] Sakcali MS, Bahadir H, Ozturk M (2008). Ecophysiology of *Capparis spinosa* L.: a plant suitable for combating desertification. Pakistan Journal of Botany.

[ref-29] Woods A, Coates KD, Hamann A (2005). Is an unprecedented *Dothistroma* needle blight epidemic related to climate change?. BioScience.

[ref-30] Yates KL, Bouchet PJ, Caley MJ, Mengersen K, Randin CF, Parnell S, Fielding AH, Bamford AJ, Ben S, Barbosa AM, Dormann CF, Elith J, Embling CB, Ervin GN, Fisher R, Gould S, Graf RF, Gregr EJ, Halpin PN, Heikkinen RK, Heinänen S, Jones AR, Krishnakumar PK, Lauria V, Lozano-Montes H, Mannocci L, Mellin C, Mesgaran MB, Moreno-Amat E, Mormede S, Novaczek E, Oppel S, Crespo GO, Peterson AT, Rapacciuolo G, Roberts JJ, Ross RE, Scales KL, Schoeman D, Snelgrove P, Sundblad G, Thuiller W, Torres LG, Verbruggen H, Wang L, Wenger S, Whittingham MJ, Zharikov Y, Zurell D, Sequeira AMM (2018). Outstanding challenges in the transferability of ecological models. Trends in Ecology & Evolution.

