# Supplementary figures and images for: Impacts of climate change on Capparis spinosa L. based on ecological niche modeling
Source: PeerJ. 2018 Oct 16;6:e5792. doi: 10.7717/peerj.5792 (PMC6195109; doi:10.7717/peerj.5792)

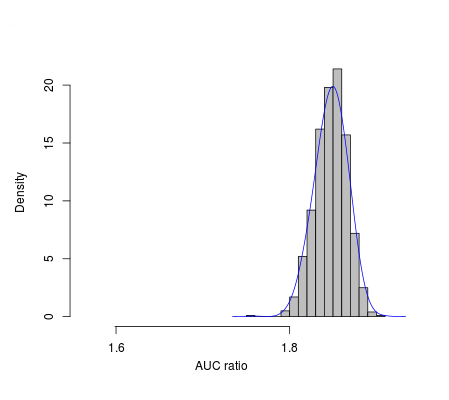

Supplement: Supplemental Information 2 [file peerj-06-5792-s002.png]
