# Supplementary material for: Impacts of climate change on Capparis spinosa L. based on ecological niche modeling
Source: PeerJ. 2018 Oct 16;6:e5792. doi: 10.7717/peerj.5792 (PMC6195109; doi:10.7717/peerj.5792)
Supplement: Supplemental Information 3 [file peerj-06-5792-s003.docx]

|  | Bio 1 | Bio 2 | Bio 3 | Bio 4 | Bio 5 | Bio 6 | Bio 7 | Bio 10 | Bio 11 | Bio 12 | Bio 13 | Bio 14 | Bio 15 | Bio 16 | Bio 17 |
| --- | --- | --- | --- | --- | --- | --- | --- | --- | --- | --- | --- | --- | --- | --- | --- |
| Bio 1 | 1.00 |  |  |  |  |  |  |  |  |  |  |  |  |  |  |
| Bio 2 | 0.36 | 1.00 |  |  |  |  |  |  |  |  |  |  |  |  |  |
| Bio 3 | 0.66 | 0.29 | 1.00 |  |  |  |  |  |  |  |  |  |  |  |  |
| Bio 4 | -0.66 | 0.01 | -0.87 | 1.00 |  |  |  |  |  |  |  |  |  |  |  |
| Bio 5 | 0.87 | 0.56 | 0.33 | -0.24 | 1.00 |  |  |  |  |  |  |  |  |  |  |
| Bio 6 | **0.94** | 0.13 | 0.77 | -0.85 | 0.68 | 1.00 |  |  |  |  |  |  |  |  |  |
| Bio 7 | -0.52 | 0.31 | -0.75 | **0.95** | -0.05 | -0.77 | 1.00 |  |  |  |  |  |  |  |  |
| Bio 10 | 0.36 | **1.00** | 0.29 | 0.01 | 0.56 | 0.13 | 0.31 | 1.00 |  |  |  |  |  |  |  |
| Bio 11 | **0.97** | 0.26 | 0.79 | -0.84 | 0.72 | **0.99** | -0.71 | 0.26 | 1.00 |  |  |  |  |  |  |
| Bio 12 | 0.05 | -0.49 | 0.31 | -0.42 | -0.24 | 0.24 | -0.53 | -0.49 | 0.18 | 1.00 |  |  |  |  |  |
| Bio 13 | 0.17 | -0.31 | 0.33 | -0.44 | -0.08 | 0.30 | -0.48 | -0.31 | 0.28 | 0.86 | 1.00 |  |  |  |  |
| Bio 14 | -0.16 | -0.50 | 0.07 | -0.14 | -0.33 | 0.01 | -0.30 | -0.50 | -0.07 | 0.68 | 0.29 | 1.00 |  |  |  |
| Bio 15 | 0.35 | 0.44 | 0.32 | -0.24 | 0.31 | 0.25 | -0.06 | 0.44 | 0.33 | -0.11 | 0.21 | -0.46 | 1.00 |  |  |
| Bio 16 | 0.15 | -0.35 | 0.32 | -0.44 | -0.11 | 0.29 | -0.49 | -0.35 | 0.26 | **0.90** | **0.99** | 0.34 | 0.16 | 1.00 |  |
| Bio 17 | -0.15 | -0.51 | 0.09 | -0.17 | -0.34 | 0.02 | -0.33 | -0.51 | -0.05 | 0.71 | 0.32 | **0.99** | -0.46 | 0.37 | 1.00 |
